# Census-based rapid and accurate metagenome taxonomic profiling

**DOI:** 10.1186/1471-2164-15-918

**Published:** 2014-10-21

**Authors:** Amirhossein Shamsaddini, Yang Pan, W Evan Johnson, Konstantinos Krampis, Mariya Shcheglovitova, Vahan Simonyan, Amy Zanne, Raja Mazumder

**Affiliations:** Department of Biochemistry and Molecular Medicine, George Washington University, Washington DC, 20037 USA; Division of Computational Biomedicine, Department of Medicine, Boston University School of Medicine, Boston, MA 02118 USA; Bioinformatics Department, The J. Craig Venter Institute, Rockville, MD 20850 USA; Department of Biological Sciences, The George Washington University, 2023 G Street NW, Washington DC, 20052 USA; Center for Biologics Evaluation and Research, Food and Drug Administration, Rockville, 20852 MD USA; Center for Conservation and Sustainable Development, Missouri Botanical Garden, St. Louis, MO 63166 USA; McCormick Genomic and Proteomic Center, George Washington University, Washington DC, 20037 USA

**Keywords:** Metagenome, Census-based, Next-gen sequence analysis, Taxonomic profiling, Diagnostics

## Abstract

**Background:**

Understanding the taxonomic composition of a sample, whether from patient, food or environment, is important to several types of studies including pathogen diagnostics, epidemiological studies, biodiversity analysis and food quality regulation. With the decreasing costs of sequencing, metagenomic data is quickly becoming the preferred typed of data for such analysis.

**Results:**

Rapidly defining the taxonomic composition (both taxonomic profile and relative frequency) in a metagenomic sequence dataset is challenging because the task of mapping millions of sequence reads from a metagenomic study to a non-redundant nucleotide database such as the NCBI non-redundant nucleotide database (nt) is a computationally intensive task. We have developed a robust subsampling-based algorithm implemented in a tool called CensuScope meant to take a ‘sneak peak’ into the population distribution and estimate taxonomic composition as if a census was taken of the metagenomic landscape. CensuScope is a rapid and accurate metagenome taxonomic profiling tool that randomly extracts a small number of reads (based on user input) and maps them to NCBI’s nt database. This process is repeated multiple times to ascertain the taxonomic composition that is found in majority of the iterations, thereby providing a robust estimate of the population and measures of the accuracy for the results.

**Conclusion:**

CensuScope can be run on a laptop or on a high-performance computer. Based on our analysis we are able to provide some recommendations in terms of the number of sequence reads to analyze and the number of iterations to use. For example, to quantify taxonomic groups present in the sample at a level of 1% or higher a subsampling size of 250 random reads with 50 iterations yields a statistical power of >99%. Windows and UNIX versions of CensuScope are available for download at https://hive.biochemistry.gwu.edu/dna.cgi?cmd=censuscope. CensuScope is also available through the High-performance Integrated Virtual Environment (HIVE) and can be used in conjunction with other HIVE analysis and visualization tools.

**Electronic supplementary material:**

The online version of this article (doi:10.1186/1471-2164-15-918) contains supplementary material, which is available to authorized users.

## Background

Metagenomics studies, where total genetic material is recovered directly from samples and sequenced, is rapidly becoming the method of choice for evaluating the taxonomic diversity of a sample. The Genomes OnLine Database (GOLD) provides information regarding genome and metagenome sequencing projects and currently lists 392 ongoing metagenomic studies that involve sequencing of 3028 samples [1]. This is an under-estimation of the actual number of ongoing projects because there are thousands of additional projects being initiated every month. A simple search in PubMed [2] of the word “metagenome” retrieves 3422 articles, of which 2205 were published within the last two years.

Metagenomics studies can range from analysis of microbial communities of marine [3] or soil [4] to microbiomes of various organs in the human body [5–7], and even to cross-biome studies [8]. Other metagenomic studies involve analysis of gene markers and genes from different species to better understand the functional components of the entire community and systems-level interactions [8–10]. Disease related studies that involve microbiome analysis using metagenomics include obesity [11–13], Crohn’s disease [14, 15], type 2 diabetes [5] and many others [16]. Such studies have vastly extended the currently available sequences in databases and will likely lead to the discovery of new genes that have useful applications in biotechnology and medicine [17, 18].

Culture-dependent survey approaches to study microbial diversity were replaced by targeted sequencing of bacterial 16S rRNA two decades ago [19]. Since then, sequencing techniques have improved and high throughput methods such as whole-genome shotgun sequencing have been used to sequence the environmental samples [20]. The cloning-dependent ‘Sanger sequencing’ was gradually replaced by a “sequencing-by-synthesis” strategy [21–23]. The advantage of this next-generation (next-gen) sequencing approach for metagenomics is that it avoids biases resulting from the cloning process used in traditional sample treatment. Thanks to the availability of these new sequencing technologies, metagenomics studies can either focus on targeted rRNA gene sequencing or whole-metagenome shotgun (WMS) sequencing. Both methods provide researchers informative data that either can be used to efficiently assess the diversity of a community or identify disease causing pathogens with increased taxonomic resolution [5, 24].

Analysis software plays an indispensable role in analyzing the components of the metagenomic data and further predicting functional attributes. In order to flexibly accommodate different scenarios and achieve different purposes, distinct approaches have been adopted by these tools. These tools utilize statistical models, like Support Vector Machines, interpolated Markov models, naïve Bayesian classifiers, etc., to train a taxonomic classifier from reference genomes and then use this classifier to cluster novel input metagenomic reads (PhyloPythiaS [25], Phymm [26], NBC: Naïve Bayes Classification tool [27]). These methods are good for analysis where sufficient prior genomic information is available. Where no prior knowledge of expected taxonomic diversity is available, running these tools becomes computationally expensive (if all reads are scanned against all known sequences) or less accurate (if reads are scanned against a signature/marker-based sequence database).

Because metagenomic reads can have multiple components, the application of universal markers can reduce the complexity and thus shrink the run time of the entire mapping process. But there are some drawbacks to this approach. This approach relies on informative slow evolving sequence features such as 16S rRNA in microorganisms or Internal Tanscribed Spacer (ITS) regions in plants to identify the community taxonomy. Many of these markers fail to distinguish between evolutionarily close yet distinct organisms. Software like AMPHORA [28] and MetaPhyler [29] were developed to analyze reads falling into certain known taxonomic clades, while MetaPhlAn concentrated on robustness of taxonomic abundance estimation based on clade specific markers present in metagenomic samples [30]. One promising tool in the area of identification of known pathogens from an environmental or clinical sample is Pathoscope [31]. Pathoscope uses a statistical framework to analyze raw next-generation sequence (NGS) reads from metagenomic samples and performs species and strain level identification based on hits to a curated signature database of known biological agents. Another strain-, species- and genus-specific genes database, CUPID, developed by our group [32] has been used by us and others to identify pathogens and related organisms from NGS data. Another approach involves construction of customized sub-databases from the NCBI-nr database [33]. The limitations of these methods are either the dependence on prior knowledge of taxonomic groups that the user is expecting or is interested in or the workflow involves iterative steps of hierarchical databases which are difficult to maintain and update on a regular basis. Additionally, the dependence on signatures which may not get amplified and sequenced for a variety of reasons [34] can result in lower accuracy.

A whole-genome analysis method serves as an emerging alternative approach that complements the marker-based methods. Unlike other previous methods, whole-genome studies require unbiased high-throughput sequencing technology and heavy computing, which in turn can provide comprehensive insights in the biodiversity and functional breadth of the organisms in the sample. This is an ideal and straightforward approach depicting the panoramic view of metagenomic data. One limitation of this method is the potential for ambiguous results due to the interruption of conserved regions by horizontally transferred sequences. However, this issue has been addressed by the lowest common ancestor (LCA) approach used by MEGAN [9] and other maximum parsimony based approaches [35]. The remaining unsolved issue is the extreme computational requirements of mapping millions of sequence reads to all known reference sequences in a reasonable amount of time. This issue becomes increasingly critical as the size of both reference databases and metagenomic datasets keep growing.

It is clear that the challenges in metagenomic analyses are many and varied [36–39]. In this paper, we focus on one of the major challenges, that is, rapidly detecting the taxonomic composition in a metagenomic short read sample with minimal computing resources. Thus, we present here an algorithm and tool which allows assessment of the biodiversity of a metagenomic sample, as while also providing a means for quality control of NGS analysis pipelines to detect contamination present in sequence reads.

## Materials and methods

### Databases and tools

The Basic Local Alignment Search Tool (Blast) formatted NCBI nt database downloaded from ftp://ftp.ncbi.nlm.nih.gov/blast/db/ is used as reference database [2]. Blast with following parameters is used: -task megablast -evalue 1e-6 -max_target_seqs 1 -best_hit_score_edge 0.05 -best_hit_overhang 0.05 -window_size 0 -perc_identity 90 [2]. Synthetic reads were generated using ART which adds plausible sequencing errors [40].

## Implementation

The graphical user interface for CensuScope is implemented using PHP and JavaScript (Additional file 1: Figure S1).

### CensuScope algorithm description and justification

Subsampling, or as it is often called subagging (*sub*sample *agg*regat*ing*), has been previously established as an efficient yet accurate method for estimating statistical quantities in complex high dimensional datasets [41]. The large-sample asymptotic properties of subsampling approaches indicate that subsampling is an efficient way to obtain highly accurate and stable estimates of statistical sampling distributions in contexts where estimates derived from the entire dataset are computationally difficult to obtain [42]. Such is the case with metagenomic samples, where the massive dataset size, combined with the ever-increasing set of reference sequence available, produce the need for highly efficient profiling tools for aligning and characterizing the population-level composition of the sample. The CensuScope subsampling approach randomly selects (with replacement) *i* samples (henceforth denoted as ‘iterations’), of size *m < <n* from the overall read dataset containing *n* total reads. From the *i* iterations, the algorithm derives point estimates as well as confidence interval estimates of abundance for each taxonomic clade at a user-defined depth. Furthermore, to remove low-proportion clades that appear due to random reads selection, we filter taxonomic groups that are not present in a majority of the subsamples based on the user defined threshold *p* (e.g. default is *p* = 80%). A high value for *p* will result in more stable results and will filter out false positive taxa that come up due to misaligned reads.

**Figure 1:**
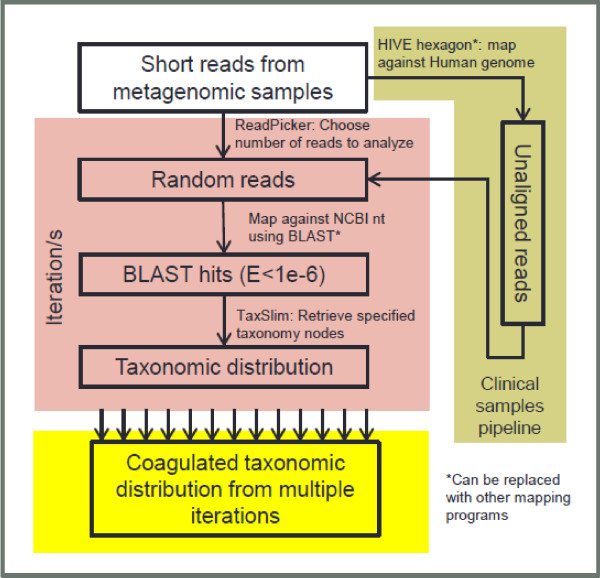
**Flowchart describing the CensuScope algorithm.** For clinical samples an additional step is required.

Figure 1 provides an overview of the CensuScope algorithm. Reads are randomly picked from the metagenomic dataset. The user can choose how many reads to pick per iteration. The number of reads picked along with the number of iterations determines the amount of time required to perform the analysis. The software also extracts taxonomic information about the hits from the NCBI taxonomy database using E-utilities (http://www.ncbi.nlm.nih.gov/books/NBK25500/). Table 1 provides details on time taken to analyze a metagenomic sample with 7.5 million reads on a personal computer (3 gigahertz (GHz) 64-bit processor; 4 gigabyte (GB) RAM). More computing power, if available, reduces run times significantly, therefore allowing selection of larger number of random reads and iterations if needed.

**Table 1:** **Time taken to analyze a metagenomic sample with one 7.5 million sequence reads on a personal computer using CensuScope**

Number of random reads	Number of iterations	Time taken (hr:min:sec)
10	10	0:35:21
100	10	0:44:57
1000	10	1:45:23

The random read picker function creates an array of pointers from locations in the dataset with a specific size relating directly to the size of metagenome. By picking *m* number of elements of this array randomly (each time picking one whole array element from a shuffle process), a dataset of randomly selected chunks of data is created. Next, the user specified number of reads are selected from the newly created dataset based on the Mersenne Twister Function (a very fast random number generator [43]).

### Definition of terms

#### Organism

The leaf-most taxonomic node in the taxonomy tree. The node can be at the species, strain, or sub-strain taxonomic level.

#### CensuScope

Census-based NGS metagenome taxonomic analyzer.

#### Best hit

Query that is listed at the top of any local alignment program. In this paper the local alignment program used is Blast.

#### Tax Slims

Conceptually similar to GO Slims (http://geneontology.org/GO.slims.shtml). Tax Slims consist of a subset of the terms in the whole taxonomy tree. Users can choose the depth of the taxonomy tree thereby creating their own Tax Slims or can choose our default Tax Slim which is set at the taxonomic depth of 3.

#### Taxonomic depth

Level of nodes within a given taxonomy tree/system.

### Sampling reads for other metagenomic pipelines

CensuScope allows users to generate a randomly picked reads set from the entire metegenome short reads file by specifying the number of short reads and iterations upon input. The short reads set can be then used by mapping/alignment algorithms. Though CensuScope is designed to analyze NGS standard output (FASTQ), the output of random picker can be either FASTA or FASTQ for user convenience as some existing algorithms can only accept FASTA files.

## Results and discussions

The two major bottlenecks in deciphering the taxonomic composition of a metagenomic sample are: a) mapping of millions of sequence reads to an exponentially growing NCBI nt; and b) creating an up-to-date index of NCBI nt for use by mapping algorithms. For the first challenge, we chose to randomly pick a limited set of reads from a metagenomic sample and map them to nt. We ran this process multiple times to identify taxonomic nodes that are hit in the majority of the iterations. For the second challenge, we chose to use a pre-computed Blast nt database that is updated regularly and distributed by NCBI.

### Statistical considerations

It is obvious that the sensitivity of the method is dependent on both the number of random reads (*m*) and the number of subsamples or iterations (*i*) that are chosen. For example, if *m* = 100 random reads and *i* = 50 subsamples are chosen, then any organism specific read which has a frequency of less than one percent will most likely be missed based on our threshold criteria: the hits to a specific organism/taxon node has to be more than *p* = 80% of the iterations. This can be problematic for clinical samples as they tend to have an over-abundance of human sequences. To avoid missing reads which are present in low frequency an additional step is included which involves mapping of the reads to the human genome first and then running CensuScope on the unaligned reads (see Figure 1 and section below on Clinical metagenome for details). This additional step can be used with any metagenomic sample where there is an abundance of reads from one specific organism and if the genome for that organism is available.

We used a simulation approach to find optimal or recommended values for the number of random reads (*m*) and the number of iterations (*i*) for multiple scenarios (see Additional file 2: Table S1a-c). We note that the estimation errors for the taxonomy proportions are directly associated with the total number of reads drawn across all iterations (i.e. *m* × *i*), whereas the margin of error for the confidence interval depends on the number of random reads. Additionally, choosing too few iterations can result in severely biased confidence intervals, usually erring by making the intervals too small. Overall, we noticed that increasing the number of random reads leads to the greatest increases in the power of detecting lower proportion taxa compared to increasing the number of intervals. Therefore, increasing the number of iterations leads to more stable and robust results that are less likely to include incorrect taxonomic groups due to misaligned reads.

Based on our simulation results, we recommend the number of iterations to be at least *i* = 50 along with *p* = 0.8. If the user wants to accurately quantify taxonomic groups present in the sample at a level of 10% or greater, we recommend a subsampling size of 25–100 random reads (see Additional file 2: Table S1a). These parameters yield a statistical power of >99% for detecting taxa present at 10%, absolute estimation error ranging from 0.003 to 0.007 and confidence interval margins of error ranging from 0.05 to 0.09. For identifying taxa present at 1% or greater, we recommend a subsampling size of 250 random reads and a sampling size of 2,500 reads for identifying taxa present at 0.1% (see Additional file 2: Table S1b-c). R code used to calculate these power results are given in Additional file 3 (censuscope.R). However, we note one limitation of this simulation study: it optimistically assumes that all reads are properly aligned to the correct source organism sequence. If there are multiple clades with closely related genomes that share sequence identity with members of the source clade, then either a follow-up step that involves mapping all reads to a select few related genomes (obtainable from genome clusters Additional file 4: Table S2) or use of a signature database that consists of species specific markers [30, 32]. The genome clusters are calculated for genomes which have a complete proteome tag in UniProt and are also tagged as complete in NCBI databases [44]. Another view of taxonomy that is rapidly gaining popularity is the concept of pan-genomes as opposed to an individual genome. For some organisms there are a set of core genes and a cloud of additional ones (found in closely related organisms/isolates) which together form the pan-genome. Such pan-genome databases can be queried by CensuScope for taxonomic profiling in addition to nt and signature databases to identify the taxonomic groups represented within a metagenomic sample.

### Test analysis results

#### Synthetic metagenome and gut microbiome

**Figure 2:**
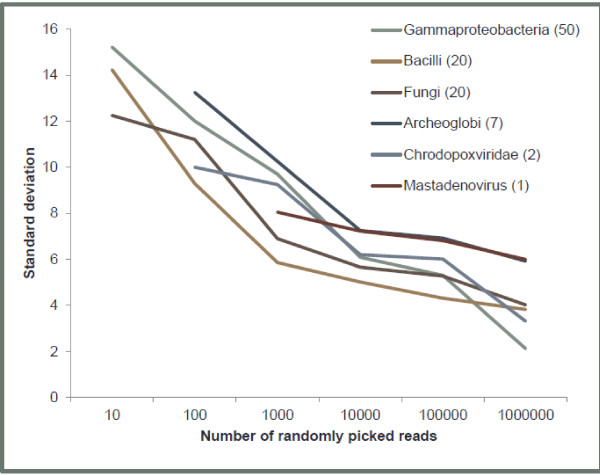
**Relationship between the numbers of reads analyzed and results obtained.** Effect of read numbers analyzed on the standard deviation of the percent of reads that are mapped to a specific taxonomic node. The numbers in parenthesis after the taxonomy node name represents the percent of reads from that taxonomy in the synthetic metagenomic sample. As expected not all taxonomy nodes are detected when the number of reads picked is low.

In order to test our algorithm, synthetic reads were generated using bacterial and viral genomes (Table 2) and the reads were mixed to create a metagenome-like sample containing 7.5 million reads. Table 2 shows that the actual content of the sample can be closely estimated using CensuScope with a fraction of the reads and minimal iterations. If the number of randomly picked reads is increased then the variation observed between each iteration decreases as can be seen in Figure 2. As expected, the number of reads that match to the correct taxonomy node decreases as we move closer to the leaf nodes. This happens because the reads hit closely related genomes. Identification of genomic neighbors is an active area of research and our group, with collaborators, has developed a method to identify genomic neighbors through the analysis of their proteomes [44]. The method is a refinement of a previous method we have used to determine the closest relative of an organism [32]. The genome clusters are calculated at different thresholds and the taxonomic ids are mapped to NCBI bioproject ids every month thereby enabling users to retrieve the closely related genomes. The results obtained from this Representative Genome and Representative Proteome project show that if we look at genomic neighbors calculated based on 75 CMT threshold (Additional file 4: Table S2), we immediately see that for organisms such as *Escherichia coli* there are other closely related genera such as *Shigella* to which the reads can also match. Therefore, at lower taxonomic nodes, the hits get split over multiple closely related organisms. For situations such as this, it is best to consider hits to all closely related organisms to make an assessment of the actual composition of the metagenome. Matching the comprehensive set of metagenomic reads to a smaller number of genomes is computationally far less challenging than matching to the entire NCBI nt. Such matching can be performed by HIVE tools (https://hive.biochemistry.gwu.edu/dna.cgi?cmd=main) or by other NGS analysis platforms such as Galaxy [45], CLC Bio (http://www.clcbio.com), Geneious (http://www.geneious.com) and others.

**Table 2:** **Synthetic metagenome original content and detected taxonomic composition**

Organism (% reads)	Tax-depth 3 ^1^(% detected ^2^)	Tax-depth 4 (% detected)	Tax-depth 5 (% detected)	Tax-depth 6 (% detected)	Tax-depth 7 (% detected)
*E. coli* (50)	γ-proteobacteria 46.7969-50.7969^3^ (48.76^4^, 48.57^5^)	Enterobacteriales 46.70-50.79 (48.74, 47.35)	Enterobacteriaceae 46.70-50.79 (48.74,47.35)	Escherichia 46.70-48.021 (47.36, 47.26)	E. coli 44.98-47.32 (46.15,47.21)
*B. subtilis* (20)	Bacilli 19.23-23.52(21.97, 19.86)	Bacillales 18.906-23.50(21.8, 19.0)	Bacillaceae 18.26-23.50(21.6, 18.90)	Bacillus 18.06-22.80(21.57, 18.1)	B. subtilis group 16.90-20.42 (21.30, 17.40)
*S. cerevisiae* (20)	Fungi 18.23-20.36 (19.29,19.07)	Dikarya 18.23-19.24 (19.05, 19.05)	Ascomycota 17.233-19.6(18.9,18.775)	Saccharomyceta 14.33-17.13(16.29, 15.92)	Saccharomycotina 14.01-15.52(15.12,15.07)
*A. fulgidus* (7)	Archaeoglobi 4.86-7.23 (6.19, 7.01)	Archaeoglobales 4.86-7.13 (6.11, 6.91)	Archaeoglobaceae 4.76-7.02 (6.09, 6.8)	Archaeoglobus 4.76-7.02 (6.09, 6.78)	A. fulgidus 3.6-6.02 (5.89, 5.80)
H. adenovirus 5 (2)	Mastadenovirus 1.23-2.14 (1.86,1.80)	Human adenovirus C 0.23-0.94 (0.18, 0.8)	Hits split^6^	Hits split	Hits spit
Vaccinia virus (1)	Chordopoxvirinae 0.98-1.83 (1.10, 1.40)	Orthopoxvirus 0.86-1.13 (1.05, 0.98)	Vaccinia virus 0.70-0.81 (0.71, 0.73)	Hits split	Hits split

^1^Refers to taxonomic depth.

^2^Percent of reads that hits the taxonomic node.

^3^Range of percent detected.

^4^Mean.

^5^Median.

^6^Hits are split between closely related organisms.

Results are based on 250 random reads and 50 iterations.

In a recent review, Segata et al. describe the current methods used to characterize microbial communities [10]. In their article they discuss methods that use intrinsic sequence properties for identification using information from sequenced microbial genome databases (extrinsic information). A popular method MetaPhlAn [30] relies on mapping of reads to a signature database that is created from 3000 sequenced microbial genomes. This means that reads not of microbial origin will not be detected. Additionally, if the signatures themselves are not sequenced then those organisms will be missed. In the following paragraphs we show why NCBI nt should be queried with CensuScope, albeit with a low number of reads to confirm MetaPhlAn results.

CensuScope uses Blast as the default mapping algorithm and NCBI nt database as the default reference database. CensuScope algorithm can also use other alignment algorithms like NGS specific alignment algorithms Bowtie2 [46] and BWA [47], and can be used to search against a curated signature database such as the MetaPhlAn db [30]. Below we provide a demonstration of how users can apply CensuScope to speed up their metagenome analysis with other pipelines using the same synthetic reads that were used for CensuScope testing as shown in Table 2. First we applied CensuScope to pick 50,000 reads randomly from the total 7.5 million synthetic reads to resize data to facilitate the comparison among different algorithms. Based on the tags in the definition line of each synthetic short read we confirmed that the composition of this randomly picked 50,000 reads is of high similarity (listed in the text below) to the total short reads pool of 7.5 million. This can be considered as a proof of the effectiveness of CensuScope’s random picker. Next the 50,000 reads were analyzed using different pipelines: A) MetaPhlAn analysis. The analysis was performed on the Galaxy version of MetaPhlAn with Bowtie2 algorithm using the ‘very sensitive local’ option. It took 8.3 s to sample the reads and 13 seconds to analyze using the Galaxy platform. The result (Additional file 5: Table S3) shows that MetaPhlAn detected 41.8% *Escherichia coli* (actual percentage 50.22%), 35.06% of the *Bacillus subtilis* (actual percentage 19.94%), 22.8% *Archaeoglobus fulgidus* (actual percentage 6.98%). The reads from eukaryota and viruses were not detected by MetaPhlAn [30] because MetaPhlAn db does not have signatures for them in their database. B) AMPHORA2 analysis. The exact same test was performed using AMPHORA [28] on their webserver AmphoraNet which uses the AMPHORA2 algorithm with the up-to-date AMPHORA markers. Due to the limited number of marker genes, the analysis resulted in no matches. C) CensuScope analysis using 50 iterations with 250 reads sampled per iteration, on the other hand, gives a result that is much closer to the actual composition of the 50,000 reads: 47.85+/-0.86% *Escherichia coli* (actual percentage 50.22%), 20.00+/-0.72% of the *Bacillus subtilis* (actual percentage 19.94%), 6.85+/-0.46% *Archaeoglobus fulgidus* (actual percentage 6.98%), and successfully identifies all the viruses and the eukaryote missed by MetaphlAn - 20.29+/-0.74% *Saccharomyces cerevisiae* (actual percentage 19.87%), 0.50+/-0.15% HAdV-C clade (actual percentage 2.05%) and 0.91+/-0.18% Vaccina virus (actual percentage 0.93%)*.* These results show that the CensuScope default method provides a comprehensive and accurate view of the taxonomic composition of a metagenomic sample.

Next, we compared our analysis method using real metagenomic data (NCBI SRA ID: SRS015989) that has been previously analyzed by the MetaPhlAn group. The results obtained from MetaPhlAn website shows that SRS015989 according to their analysis consists of Actinomycetales 43.83%, Neisseriales 20.37%, Lactobacillales 12.81%, Flavobacteriales 10.47%, Pasteurellales 6.44% and Selenomonadales 2.21%. Our analysis using CensuScope with suggested parameters (250 randomly picked reads and 50 iterations) provided the following results: Actinomycetales 42.58+/-1.73%, Neisseriales 3.95+/-0.57%, Lactobacillales 30.14+/-1.57%, Flavobacteriales 3.20+/-0.47%, Pasteurellales 9.31+/-0.90%, Selenomonadales 2.54+/-0.47% and Fusobacteriales 0.77+/-0.27%. Additionally artificial sequence vectors (2.03+/-0.43%) and eukaryotic sequences (1.79+/- 0.39%) were also reported. To obtain a reference composition of this real metagenome for our comparison, we randomly picked 10,000 reads by using the same method described in the previous section. It is interesting to note that MetaPhlAn analysis (http://huttenhower.sph.harvard.edu/content/metaphlan-galaxy) of randomly picked 10,000 reads from SRS015989 identifies Actinomycetales 84.26% and Neisseriales 15.73% and nothing else. Blast against NCBI nt analysis of these 10,000 reads provides the following results: Actinomycetales 41.25%, Neisseriales 3.87%, Flavobacteriales 2.45%, Lactobacillales 28.49%, Pasteurellales 8.98%, Selenomonadales 2.62% which closely resembles CensuScope results using default parameters analyzing the same 10,000 reads (Actinomycetales 40.97+/-1.32%, Neisseriales 4.35+/-0.53%, Flavobacteriales 4.45+/-0.53%, Lactobacillales 30.64+/-1.68%, Pasteurellales 9.62+/-0.84%, Selenomonadales 2.86+/-0.49%).

Motivated by the significant difference of the detected taxonomic abundance between the results generated by MetaphlAn and CensuScope using the same reads set, we performed an additional analysis on a relatively small but complete real metagenome dataset (SRS011236). As this metagenomic data is only 109 MB (filtered and trimmed by Human Microbiome Project (HMP) group), Blast against NCBI nt results using all the reads can be easily used to provide us with a reference composition. Based on the HMP paper, the exact same short reads file has been directly analyzed by MetaphlAn to produce the result which contains only three clades: 69.87% Actinomycetales, 1.28% Bacillales and 28.85% Clostridiales (Additional file 6: Table S4). The Blast analysis using the entire dataset (called ‘Blast result’ from here onwards) and CensuScope analysis of the same metagenome provides an overall similar taxonomic composition that is distinct from what is presented by MetaphlAn. CensuScope (250 random reads/50 iterations) found 17.83+/-0.94% Actinomycetales (Blast result 17.99%), 0.48+/-0.16% Bacillales (Blast result 0.57%) and 0.60+/-0.17% Clostridiales (Blast result 0.56%). In addition CensuScope and Blast results identified several other clades that are not detected by MetaPhlAn. The novel detected clades include 2.97+/- 0.36% Lactobacillales (Blast result 3.03%), 35.07+/-1.43% Homininae (Blast result 35.59%), 1.16+/-0.25% Siphoviridae (Blast result 1.05%), etc. (Additional file 6: Table S4).

#### Fungal metagenome

**Figure 3:**
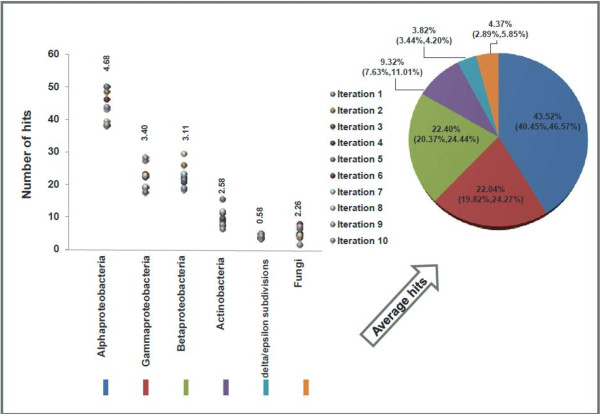
**Metagenomic analysis results of sample collected from decaying wood.** One thousand reads were randomly picked and analyzed using CensuScope with the number of iterations set at 10. The number above the dots represents standard deviation. The pie chart shows the average distribution of hits in the different taxonomic nodes with mean and confidence intervals in parenthesis.

Identification and characterization of organisms that degrade wood are active areas of research [48, 49]. It is well known that many fungal species are involved in the degradation of wood. More recently it has been recognized that bacteria might collaboratively work with fungal species to accelerate wood decay [50]. Emerging molecular techniques are expanding our knowledge of the diversity of wood inhabiting organisms [51, 52]. To better understand the biodiversity of wood decomposing organisms, environmental DNA was extracted from two decaying logs and used to generate 50 base pair single-end reads on the Illumina HiSeq. Given the accepted prevalence of fungi as wood decayers, we hypothesized that the majority of the 1.1 million reads in the metagenomic dataset would belong to fungi with a smaller fraction of the reads belonging to bacteria. CensuScope analysis, using Blast with E-value threshold of 1e-6, number of random reads selected per iteration set to 10,000 and number of iterations set to 10, found that the majority of reads are from proteobacteria and only 4.37% of the reads are from fungi (Figure 3). Proteobacteria have been found to be highly abundant in decaying wood [51] and are known to be capable of lignin decomposition [53]. The low percentage of fungi and prevalence of bacteria in this community could be due to either few fungal species truly present in the examined samples or lack of appropriate fungal sequences in the NCBI nt database. It is clear that more samples would need to be collected and analyzed to better understand the role of bacteria and fungi in wood degradation.

#### Clinical metagenome (respiratory tract infection)

**Figure 4:**
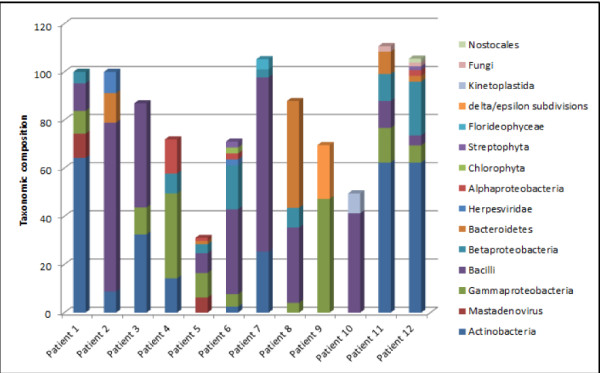
**Metagenomic analysis of samples isolated from patients suffering from upper respiratory tract infection.** Analysis results after filtering out Human specific reads from 12 patients (1000 reads/iteration; 10 iterations). Only hits above one percent are shown.

Characterizing the microbiome in hard to treat respiratory tract infections is critical for disease treatment and management [54]. CensuScope analysis of a respiratory tract metagenome containing 3.2 million reads, using Blast with E-value threshold of 1e-6, number of random reads selected per iteration set to 10,000 and the number of iteration set to 10 reveals that 99.7% of reads are from *Homo sapiens* and ~0.08% of the reads are from Bacilli (data not shown). An overabundance of reads from the host is expected in clinical samples. Using an additional step that includes mapping the metagenomic reads to the human genome and then aligning the unaligned reads to NCBI nt gives a much better resolution as can be seen in Figure 4 for twelve patient samples. The initial reads were mapped to the human genome (minimum match length 80%; mismatch allowed 10%) using HIVE hexagon [55] and the unaligned reads were downloaded (Additional file 1: Figures S1 and S2) and CensuScope was run to obtain the taxonomic composition of the non-human reads.

It is always possible that the best hit of a read can be to organisms from which the reads did not originate. This is inevitable if there are identical or closely related sequences in multiple organisms. Therefore, to attain strain level identification one can envision a step where the reads are run through a database of sequence signatures similar to a methodology that we have described previously [32]. One caveat is that it is possible that unique signatures may not have been sequenced in the metagenomic sample. Therefore, to obtain strain or isolate level identification, standard widely used follow-up experiments can be performed [56, 57] or alternatively, the entire metagenome can be mapped to all known sequences under the specific taxonomy node. Algorithms to create such pan-genomes are already available [58–60] and can be implemented into the diagnostic workflow for closely related genomes.

### Usage instruction

UNIX and Windows versions are available for download from the HIVE website (http://hive.biochemistry.gwu.edu). The download file is a zip folder of approximately 10GB size. The download package contains the CensuScope program, the indexed concatenated NCBI nt Blast database (reference database) and two sample metagenome datasets (virus.fastq and prok.fastq).

Specific instructions are as follows: For Windows: Run CensuScope.exe; choose the path to the metagnome file (e.g. c:\CensuScope\source\virus.fastq); set the number random reads; set the number of iterations; click submit. The output will be generated in \CensuScope\sample\. For UNIX: Install PHP (if not already installed) from http://php.net/downloads.php; provide arguments -s: number of random reads, -i: number of iterations, -t: depth of taxonomy tree to classify the reads into (default setting is 3), -d: metagenome file name, -p: CensuScope package path. Example command:

$php CensuScope.php -i 10 -s 1000 -t 3 -d ‘/user/desktop/fungi.fastq’ -p ‘/user/package’. Internet access is required to run the program. Additional instruction on how to use CensuScope is available in file Additional file 7.

### Explanation of results

CensuScope is designed and optimized for rapid analyses of NGS metagenomic data and provids users with standard formatted reports of hits classified into species or higher taxonomic nodes. There are four output files: 1) log.txt - this text file provides all parameters which was used to run and generate the result. 2) gi_centric_table.csv – NCBI gi numbers sorted result. 3) tax_centric_table.csv - taxonomy id sorted result. 4) taxslim_centric_table.csv - user defined taxonomy nodes sorted result. The table also includes information on the number of times a specific taxnode was hit. This information provides an estimate of the reliability of taxonomic distribution that is reported.

### CensuScope on High-performance Integrated Virtual Environment (HIVE)

CensuScope has been parallelized in HIVE. The HIVE CensuScope tool determines the taxonomic composition of a metagenomic sample by analyzing the sequence data through rapid iterative mapping to all known sequences using Blast.. Once the data is loaded into HIVE using HIVE’s dmDownloader utility (see “HIVE Downloader Tutorial” available in main pages of HIVE website) it is available for analysis. CensuScope can be used for quick sample origin discovery, to study metagenomic samples or mixed viral populations, or to evaluate the possible contamination of samples. CensuScope result is downloadable in Scalable Vector Graphics (SVG) format for visualization and Comma-separated Values (CSV) format for the mapping results. Detailed step-by-step instruction is available in Additional file 7.

## Conclusion

The three key elements required in the analysis of metagenomic samples to obtain the taxonomic composition are: a) a comprehensive non-redundant reference sequence database coupled with b) a generally accepted taxonomy of known organisms, and c) the alignment software for sequence comparison. The first element consists of public sequence databases, which are maintained by NCBI, ENA and DDBJ through the International Nucleotide Sequence Database Collaboration (INSDC) [61]. The content of nt is heavily biased towards organisms that can be cultivated and organisms that have socio-economic benefits. It is expected that over time this bias will decrease as more organisms are cultured and sequencing techniques that do not require culturing gets easier and cheaper to use. The second component, the sequence alignment tool, is another critical step that determines the computational cost of analysis. Fast mapping algorithms, longer reads, and availability of mapping ready indexed reference sequence databases is expected to lower the barrier for this step. The third component is the taxonomic classification of species used which is also evolving over time. Our approach is based on the NCBI taxonomic system, which is maintained and updated by a team of taxonomy experts [2] and, importantly, linked directly to the molecular sequence data. Complementary advances which cluster genomes to create pan-genome sequences is expected to provide better resolution where the taxonomy tree does not correctly reflect the ‘sequence space cloud’ for groups of closely related organisms. CensuScope being highly flexible can be easily modified to use any indexed sequence database or taxonomic classification or mapping algorithm. For example, users can download a signature database such as MetaPhlAn or AMPHORA and use CensuScope to query that database resulting in even faster performance. It is possible that some organisms can be missed using signature databases such as MetaPhlAn or AMPHORA. MetaPhlAn consists of unique clade-specific marker genes identified from 3,000 reference genomes [30] and AMPHORA2 uses 31 bacterial markers and additional markers from 50 archaeal genomes [62]. Additionally, it is also possible that the metagenomic sequencing process may not result in the sequencing of the markers. Therefore, it is desirable to first use CensuScope with NCBI nt to get a comprehensive overview prior to using signature databases. Finally, to identify organisms which are in low abundance in the metagenomic sample users should filter out reads from highly represented organisms using NGS alignment tools such as HIVE Hexagon [55], Bowtie [46] etc. and then use CensuScope.

Based on user requests, we will continue to expand CensuScope capabilities based on the above mentioned considerations.

## Availability

CensuScope is freely available for download from: https://hive.biochemistry.gwu.edu/dna.cgi?cmd=censuscope. To run CensuScope on the HIVE Cloud servers users are required to register.

## Authors’ information

Amirhossein Shamsaddini and Yang Pan should be considered as first authors.

## Electronic supplementary material

Additional file 1: Figure S1: Snapshot of CensuScope interface. **Figure S2.** Snapshot of HIVE Hexagon (short read mapping tool) input and results interface. Details on HIVE Hexagon is available at http://hive.biochemistry.gwu.edu/HIVE_AlgorithmicsPoster.pdf. (PPTX 1 MB)

Additional file 2: Table S1: Simulation results for detection and estimation of a taxonomic clade that is present at a level of 0.1%, 1% and 10% in the sample. (DOCX 22 KB)

Additional file 3: **R code used to calculate the power results (censuscope.R).** (ZIP 670 bytes)

Additional file 4: Table S2: Genomic neighbors calculated based on 75 CMT threshold obtained from Representative Genome and Representative Proteome. (XLSX 782 KB)

Additional file 5: Table S3: Result from MetaPhlAn-Galaxy version on analysis of 50,000 reads from synthetic test data. (XLSX 9 KB)

Additional file 6: Table S4: Comparison between BLAST, CensuScope, and MetaPhlAn result using a real metagenomic data (SRS011236). (XLSX 14 KB)

Additional file 7: **HIVE CensuScope Tutorial.** (DOCX 608 KB)

## References

[1] Pagani I, Liolios K, Jansson J, Chen IM, Smirnova T, Nosrat B, Markowitz VM, Kyrpides NC (2012). The Genomes OnLine Database (GOLD) v.4: status of genomic and metagenomic projects and their associated metadata. Nucleic Acids Res.

[2] NCBI_Resource_Coordinators (2013). Database resources of the national center for biotechnology information. Nucleic Acids Res.

[3] Kennedy J, Flemer B, Jackson SA, Lejon DP, Morrissey JP, O’Gara F, Dobson AD (2010). Marine metagenomics: new tools for the study and exploitation of marine microbial metabolism. Mar Drugs.

[4] Bru D, Ramette A, Saby NP, Dequiedt S, Ranjard L, Jolivet C, Arrouays D, Philippot L (2011). Determinants of the distribution of nitrogen-cycling microbial communities at the landscape scale. ISME J.

[5] Qin J, Li R, Raes J, Arumugam M, Burgdorf KS, Manichanh C, Nielsen T, Pons N, Levenez F, Yamada T, Mende DR, Li J, Xu J, Li S, Li D, Cao J, Wang B, Liang H, Zheng H, Xie Y, Tap J, Lepage P, Bertalan M, Batto JM, Hansen T, Le Paslier D, Linneberg A, Nielsen HB, Pelletier E, Renault P (2010). A human gut microbial gene catalogue established by metagenomic sequencing. Nature.

[6] Human_Microbiome_Project_Consortium (2012). Structure, function and diversity of the healthy human microbiome. Nature.

[7] Greenblum S, Turnbaugh PJ, Borenstein E (2012). Metagenomic systems biology of the human gut microbiome reveals topological shifts associated with obesity and inflammatory bowel disease. Proc Natl Acad Sci U S A.

[8] Fierer N, Leff JW, Adams BJ, Nielsen UN, Bates ST, Lauber CL, Owens S, Gilbert JA, Wall DH, Caporaso JG (2012). Cross-biome metagenomic analyses of soil microbial communities and their functional attributes. Proc Natl Acad Sci U S A.

[9] Huson DH, Auch AF, Qi J, Schuster SC (2007). MEGAN analysis of metagenomic data. Genome Res.

[10] Segata N, Boernigen D, Tickle TL, Morgan XC, Garrett WS, Huttenhower C (2013). Computational meta’omics for microbial community studies. Mol Syst Biol.

[11] Backhed F, Ding H, Wang T, Hooper LV, Koh GY, Nagy A, Semenkovich CF, Gordon JI (2004). The gut microbiota as an environmental factor that regulates fat storage. Proc Natl Acad Sci U S A.

[12] Turnbaugh PJ, Hamady M, Yatsunenko T, Cantarel BL, Duncan A, Ley RE, Sogin ML, Jones WJ, Roe BA, Affourtit JP, Egholm M, Henrissat B, Heath AC, Knight R, Gordon JI (2009). A core gut microbiome in obese and lean twins. Nature.

[13] Kau AL, Ahern PP, Griffin NW, Goodman AL, Gordon JI (2011). Human nutrition, the gut microbiome and the immune system. Nature.

[14] Manichanh C, Rigottier-Gois L, Bonnaud E, Gloux K, Pelletier E, Frangeul L, Nalin R, Jarrin C, Chardon P, Marteau P, Roca J, Dore J (2006). Reduced diversity of faecal microbiota in Crohn’s disease revealed by a metagenomic approach. Gut.

[15] Morgan XC, Tickle TL, Sokol H, Gevers D, Devaney KL, Ward DV, Reyes JA, Shah SA, LeLeiko N, Snapper SB, Bousvaros A, Korzenik J, Sands BE, Xavier RJ, Huttenhower C (2012). Dysfunction of the intestinal microbiome in inflammatory bowel disease and treatment. Genome Biol.

[16] Blumberg R, Powrie F (2012). Microbiota, disease, and back to health: a metastable journey. Sci Transl Med.

[17] Steele HL, Streit WR (2005). Metagenomics: advances in ecology and biotechnology. FEMS Microbiol Lett.

[18] Wooley JC, Godzik A, Friedberg I (2010). A primer on metagenomics. PLoS Comput Biol.

[19] Schmidt TM, DeLong EF, Pace NR (1991). Analysis of a marine picoplankton community by 16S rRNA gene cloning and sequencing. J Bacteriol.

[20] Venter JC, Remington K, Heidelberg JF, Halpern AL, Rusch D, Eisen JA, Wu D, Paulsen I, Nelson KE, Nelson W, Fouts DE, Levy S, Knap AH, Lomas MW, Nealson K, White O, Peterson J, Hoffman J, Parsons R, Baden-Tillson H, Pfannkoch C, Rogers YH, Smith HO (2004). Environmental genome shotgun sequencing of the Sargasso Sea. Science.

[21] Meldrum D (2000). Automation for genomics, part one: preparation for sequencing. Genome Res.

[22] Margulies M, Egholm M, Altman WE, Attiya S, Bader JS, Bemben LA, Berka J, Braverman MS, Chen YJ, Chen Z (2005). Genome sequencing in microfabricated high-density picolitre reactors. Nature.

[23] Zhang K, Martiny AC, Reppas NB, Barry KW, Malek J, Chisholm SW, Church GM (2006). Sequencing genomes from single cells by polymerase cloning. Nat Biotechnol.

[24] Tyson GW, Chapman J, Hugenholtz P, Allen EE, Ram RJ, Richardson PM, Solovyev VV, Rubin EM, Rokhsar DS, Banfield JF (2004). Community structure and metabolism through reconstruction of microbial genomes from the environment. Nature.

[25] Patil KR, Roune L, McHardy AC (2012). The PhyloPythiaS web server for taxonomic assignment of metagenome sequences. PLoS One.

[26] Brady A, Salzberg S (2011). PhymmBL expanded: confidence scores, custom databases, parallelization and more. Nat Methods.

[27] Rosen GL, Reichenberger ER, Rosenfeld AM (2011). NBC: the Naive Bayes Classification tool webserver for taxonomic classification of metagenomic reads. Bioinformatics.

[28] Wu M, Scott AJ (2012). Phylogenomic analysis of bacterial and archaeal sequences with AMPHORA2. Bioinformatics.

[29] Liu B, Gibbons T, Ghodsi M, Treangen T, Pop M (2011). Accurate and fast estimation of taxonomic profiles from metagenomic shotgun sequences. BMC Genomics.

[30] Segata N, Waldron L, Ballarini A, Narasimhan V, Jousson O, Huttenhower C (2012). Metagenomic microbial community profiling using unique clade-specific marker genes. Nat Methods.

[31] Francis OE, Bendall M, Manimaran S, Hong C, Clement NL, Castro-Nallar E, Snell Q, Schaalje GB, Clement MJ, Crandall KA, Johnson WE (2013). Pathoscope: Species identification and strain attribution with unassembled sequencing data. Genome Res.

[32] Mazumder R, Natale DA, Murthy S, Thiagarajan R, Wu CH (2005). Computational identification of strain-, species- and genus-specific proteins. BMC bioinformatics.

[33] Yu K, Zhang T (2013). Construction of customized sub-databases from NCBI-nr database for rapid annotation of huge metagenomic datasets using a combined BLAST and MEGAN approach. PLoS One.

[34] Abbai NS, Govender A, Shaik R, Pillay B (2012). Pyrosequence analysis of unamplified and whole genome amplified DNA from hydrocarbon-contaminated groundwater. Mol Biotechnol.

[35] Berger SA, Stamatakis A (2011). Aligning short reads to reference alignments and trees. Bioinformatics.

[36] Teeling H, Glockner FO (2012). Current opportunities and challenges in microbial metagenome analysis–a bioinformatic perspective. Brief Bioinform.

[37] Hunter CI, Mitchell A, Jones P, McAnulla C, Pesseat S, Scheremetjew M, Hunter S (2012). Metagenomic analysis: the challenge of the data bonanza. Brief Bioinform.

[38] Mande SS, Mohammed MH, Ghosh TS (2012). Classification of metagenomic sequences: methods and challenges. Brief Bioinform.

[39] Prakash T, Taylor TD (2012). Functional assignment of metagenomic data: challenges and applications. Brief Bioinform.

[40] Huang W, Li L, Myers JR, Marth GT (2012). ART: a next-generation sequencing read simulator. Bioinformatics.

[41] Bühlmann P, Yu B (2002). Analyzing Bagging. Ann Stat.

[42] DN P, JP R (1994). Large sample confidence regions based on subsamples under minimal assumptions. Annals of Statistics.

[43] Matsumoto M, Mersenne Twister NT (1998). Mersenne Twister: A 623-dimensionally equidistributed uniform pseudorandom number generator. ACM Trans Model Comput Simul.

[44] Chen C, Natale DA, Finn RD, Huang H, Zhang J, Wu CH, Mazumder R (2011). Representative proteomes: a stable, scalable and unbiased proteome set for sequence analysis and functional annotation. PLoS One.

[45] Giardine B, Riemer C, Hardison RC, Burhans R, Elnitski L, Shah P, Zhang Y, Blankenberg D, Albert I, Taylor J, Miller W, Kent WJ, Nekrutenko A (2005). Galaxy: a platform for interactive large-scale genome analysis. Genome Res.

[46] Langmead B, Salzberg SL (2012). Fast gapped-read alignment with Bowtie 2. Nat Methods.

[47] Li H, Durbin R (2009). Fast and accurate short read alignment with Burrows-Wheeler transform. Bioinformatics.

[48] Kaffenberger JT, Schilling JS (2013). Using a grass substrate to compare decay among two clades of brown rot fungi. Appl Microbiol Biotechnol.

[49] Morel M, Meux E, Mathieu Y, Thuillier A, Chibani K, Harvengt L, Jacquot JP, Gelhaye E (2013). Xenomic networks variability and adaptation traits in wood decaying fungi. J Microbial Biotechnol.

[50] Kamei I, Yoshida T, Enami D, Meguro S (2012). Coexisting Curtobacterium bacterium promotes growth of white-rot fungus Stereum sp. Curr Microbiol.

[51] Zhang HB, Yang MX, Tu R (2008). Unexpectedly high bacterial diversity in decaying wood of a conifer as revealed by a molecular method. Int Biodeter Biodegr.

[52] Kubartova A, Ottosson E, Dahlberg A, Stenlid J (2012). Patterns of fungal communities among and within decaying logs, revealed by 454 sequencing. Mol Ecol.

[53] Bugg TD, Ahmad M, Hardiman EM, Singh R (2011). The emerging role for bacteria in lignin degradation and bio-product formation. Curr Opin Biotechnol.

[54] Lysholm F, Wetterbom A, Lindau C, Darban H, Bjerkner A, Fahlander K, Lindberg AM, Persson B, Allander T, Andersson B (2012). Characterization of the viral microbiome in patients with severe lower respiratory tract infections, using metagenomic sequencing. PLoS One.

[55] Santana-Quintero L, Dingerdissen H, Thierry-Mieg J, Mazumder R, Simonyan V (2014). HIVE-hexagon: high-performance, parallelized sequence alignment for next-generation sequencing data analysis. PLoS One.

[56] Krishna NK, Cunnion KM (2012). Role of molecular diagnostics in the management of infectious disease emergencies. Med Clin North Am.

[57] Sibley CD, Peirano G, Church DL (2012). Molecular methods for pathogen and microbial community detection and characterization: current and potential application in diagnostic microbiology. Infect Genet Evol.

[58] Mann RA, Smits TH, Buhlmann A, Blom J, Goesmann A, Frey JE, Plummer KM, Beer SV, Luck J, Duffy B, Rodoni B (2013). Comparative genomics of 12 strains of Erwinia amylovora identifies a pan-genome with a large conserved core. PLoS One.

[59] Fouts DE, Brinkac L, Beck E, Inman J, Sutton G (2012). PanOCT: automated clustering of orthologs using conserved gene neighborhood for pan-genomic analysis of bacterial strains and closely related species. Nucleic Acids Res.

[60] Zhao Y, Wu J, Yang J, Sun S, Xiao J, Yu J (2012). PGAP: pan-genomes analysis pipeline. Bioinformatics.

[61] Karsch-Mizrachi I, Nakamura Y, Cochrane G (2012). The international nucleotide sequence database collaboration. Nucleic Acids Res.

[62] Wu M, Eisen JA (2008). A simple, fast, and accurate method of phylogenomic inference. Genome Biol.

