# Supplementary material for: Census-based rapid and accurate metagenome taxonomic profiling
Source: BMC Genomics. 2014 Oct 21;15(1):918. doi: 10.1186/1471-2164-15-918 (PMC4218995; doi:10.1186/1471-2164-15-918)
Supplement: Supplementary file 1 — Additional file 1: Figure S1: Snapshot of CensuScope interface. Figure S2. Snapshot of HIVE Hexagon (short read mapping tool) input and results interface. Details on HIVE Hexagon is available at http://hive.biochemistry.gwu.edu/HIVE_AlgorithmicsPoster.pdf. (PPTX 1 MB) [file 12864_2013_6618_MOESM1_ESM.pptx]

## Slide 1
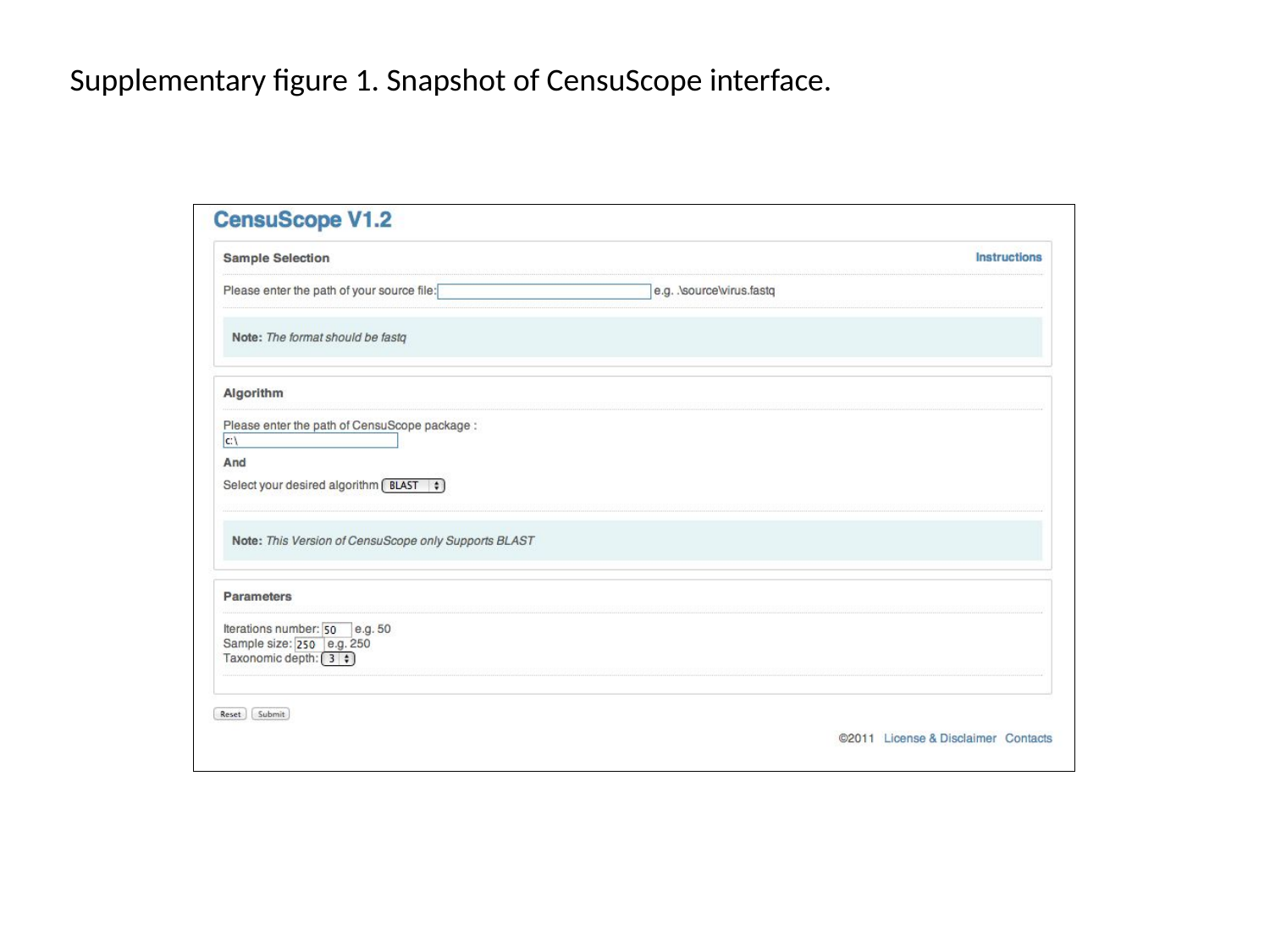

Supplementary figure 1. Snapshot of CensuScope interface.

## Slide 2
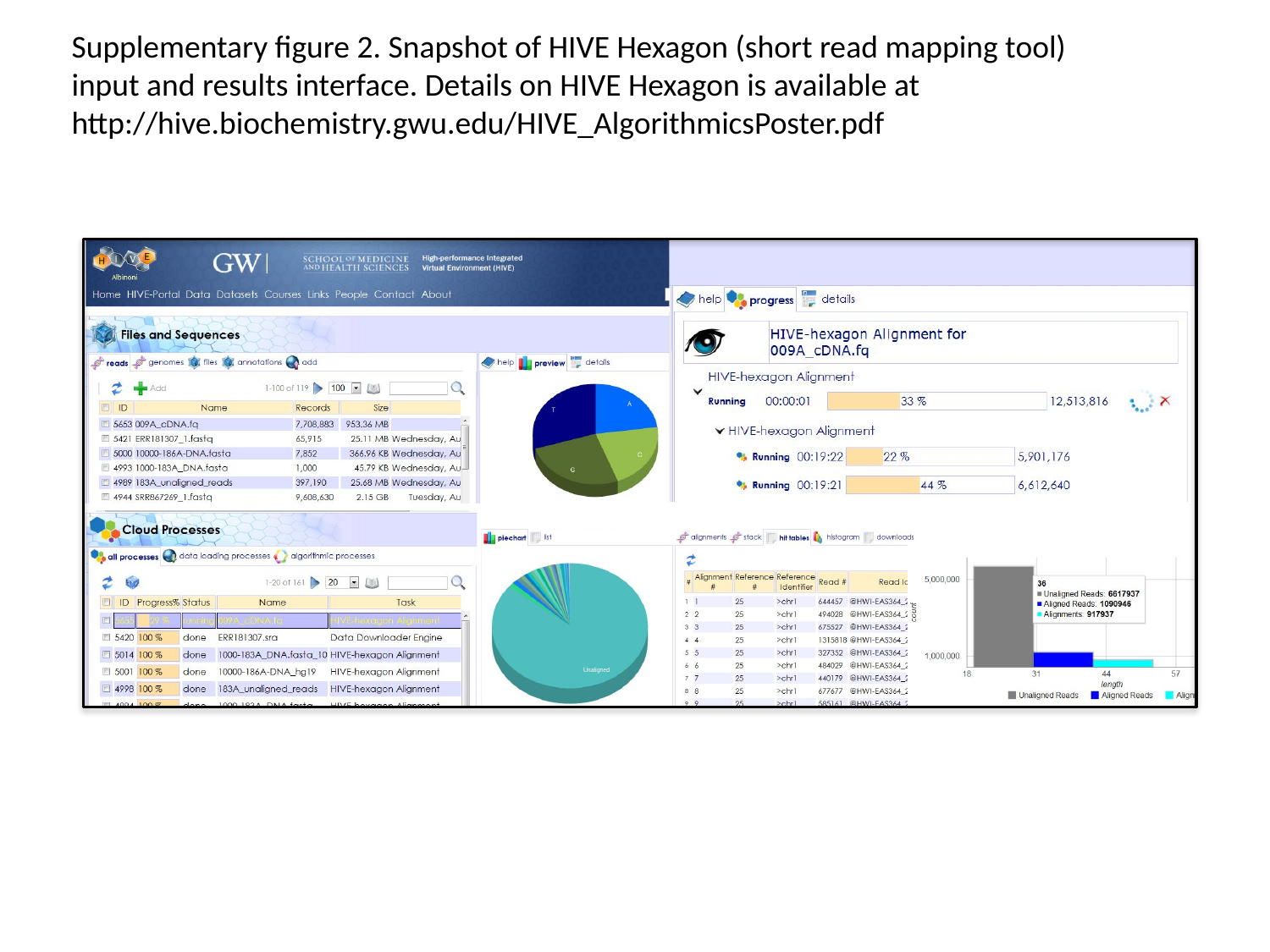

Supplementary figure 2. Snapshot of HIVE Hexagon (short read mapping tool)
input and results interface. Details on HIVE Hexagon is available at
http://hive.biochemistry.gwu.edu/HIVE_AlgorithmicsPoster.pdf
