# Supplementary material for: Census-based rapid and accurate metagenome taxonomic profiling
Source: BMC Genomics. 2014 Oct 21;15(1):918. doi: 10.1186/1471-2164-15-918 (PMC4218995; doi:10.1186/1471-2164-15-918)
Supplement: Supplementary file 2 — Additional file 2: Table S1: Simulation results for detection and estimation of a taxonomic clade that is present at a level of 0.1%, 1% and 10% in the sample. (DOCX 22 KB) [file 12864_2013_6618_MOESM2_ESM.docx]

**Supplemental Table 1a.** Simulation results for detection and estimation of a taxonomic clade that is present at a level of 10% in the sample.

|  |  |  | **Number of Reads Sampled** | | |
| --- | --- | --- | --- | --- | --- |
|  |  |  | **25** | **100** | **250** |
| **Number of Iterations** | **10** | **Detection Power (%)** | 93.8 | 99.7 | 100 |
|  |  | **Estimation Error (%)** | 15.5 | 7.7 | 4.9 |
|  |  | **Confidence Interval** | (0.03, 0.18) | (0.06, 0.14) | (0.08, 0.13) |
|  |  |  |  |  |  |
|  | **50** | **Detection Power (%)** | 99.8 | 100 | 100 |
|  |  | **Estimation Error (%)** | 6.5 | 3.3 | 2.1 |
|  |  | **Confidence Interval** | (0.02, 0.20) | (0.06, 0.15) | (0.07, 0.13) |
|  |  |  |  |  |  |
|  | **100** | **Detection Power (%)** | 100 | 100 | 100 |
|  |  | **Estimation Error (%)** | 5.0 | 2.4 | 1.5 |
|  |  | **Confidence Interval** | (0.01, 0.20) | (0.05, 0.15) | (0.07, 0.13) |

**Supplemental Table 1b.** Simulation results for detection and estimation of a taxonomic clade that is present at a level of 1% in the sample.

|  |  |  | **Number of Reads Sampled** | | | |
| --- | --- | --- | --- | --- | --- | --- |
|  |  |  | **100** | **250** | **500** | **1000** |
| **Number of Iterations** | **10** | **Detection Power (%)** | 20.5 | 95.9 | 100 | 100 |
|  |  | **Estimation Error (%)** | 24.3 | 16.0 | 11.2 | 7.9 |
|  |  | **Confidence Interval** | (0.000, 0.024) | (0.003, 0.019) | (0.005, 0.016) | (0.006, 0.014) |
|  |  |  |  |  |  |  |
|  | **50** | **Detection Power (%)** | 1.0 | 99.9 | 100 | 100 |
|  |  | **Estimation Error (%)** | 10.8 | 7.2 | 5.0 | 3.4 |
|  |  | **Confidence Interval** | (0.000, 0.027) | (0.001, 0.021) | (0.004, 0.017) | (0.005, 0.015) |
|  |  |  |  |  |  |  |
|  | **100** | **Detection Power (%)** | 0.0 | 100 | 100 | 100 |
|  |  | **Estimation Error (%)** | 8.2 | 4.9 | 3.7 | 2.5 |
|  |  | **Confidence Interval** | (0.000, 0.028) | (0.001, 0.021) | (0.004, 0.017) | (0.005, 0.015) |
|  |  |  |  |  |  |  |
|  | **500** | **Detection Power (%)** | 0.0 | 100 | 100 | 100 |
|  |  | **Estimation Error (%)** | 3.6 | 2.3 | 1.6 | 1.1 |
|  |  | **Confidence Interval** | (0.000, 0.030) | (0.000, 0.021) | (0.004, 0.018) | (0.005, 0.015) |

**Supplemental Table 1c.** Simulation results for detection and estimation of a taxonomic clade that is present at a level of 0.1% in the sample.

|  |  |  | **Number of Reads Sampled** | | | | | |
| --- | --- | --- | --- | --- | --- | --- | --- | --- |
|  |  |  | **100** | **250** | **500** | **1000** | **2500** | **10000** |
| **Number of Iterations** | **10** | **Detection Power (%)** | 0.0 | 0.0 | 1.0 | 22.1 | 94.3 | 100 |
|  |  | **Estimation Error (%)** | 77.0 | 51.7 | 35.7 | 25.0 | 15.8 | 7.9 |
|  |  | **Confidence Interval** | (0.000, 0.005) | (0.000, 0.004) | (0.000, 0.003) | (0.000, 0.002) | (0.000, 0.002) | (0.001, 0.001) |
|  |  |  |  |  |  |  |  |  |
|  | **50** | **Detection Power (%)** | 0.0 | 0.0 | 0.0 | 0.7 | 100 | 100 |
|  |  | **Estimation Error (%)** | 35.0 | 22.5 | 15.4 | 10.8 | 6.9 | 3.4 |
|  |  | **Confidence Interval** | (0.000, 0.008) | (0.000, 0.004) | (0.000, 0.004) | (0.000, 0.003) | (0.000, 0.002) | (0.000, 0.002) |
|  |  |  |  |  |  |  |  |  |
|  | **100** | **Detection Power (%)** | 0.0 | 0.0 | 0.0 | 0.0 | 100 | 100 |
|  |  | **Estimation Error (%)** | 25.0 | 15.9 | 11.1 | 7.9 | 5.0 | 2.5 |
|  |  | **Confidence Interval** | (0.000, 0.009) | (0.000, 0.004) | (0.000, 0.004) | (0.000, 0.003) | (0.000, 0.002) | (0.000, 0.002) |
|  |  |  |  |  |  |  |  |  |
|  | **500** | **Detection Power (%)** | 0.0 | 0.0 | 0.0 | 0.0 | 100 | 100 |
|  |  | **Estimation Error (%)** | 11.0 | 7.1 | 5.0 | 3.7 | 2.3 | 1.1 |
|  |  | **Confidence Interval** | (0.000, 0.010) | (0.000, 0.004) | (0.000, 0.004) | (0.000, 0.003) | (0.000, 0.002) | (0.000, 0.002) |
