# Supplementary material for: Census-based rapid and accurate metagenome taxonomic profiling
Source: BMC Genomics. 2014 Oct 21;15(1):918. doi: 10.1186/1471-2164-15-918 (PMC4218995; doi:10.1186/1471-2164-15-918)
Supplement: Supplementary file 7 — Additional file 7: HIVE CensuScope Tutorial. (DOCX 608 KB) [file 12864_2013_6618_MOESM7_ESM.docx]

**
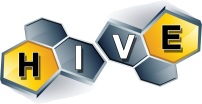
 HIVE CensuScope Tutorial**

The purpose of this tutorial is to guide the user through the process of analyzing a metagenomic sample using the parallelized Cloud version of the **CensuScope** tool.

**TABLE OF CONTENTS**

**Introduction**

**1. Selecting Inputs**

**2. Input Parameters**

**2.1** Most frequently modified parameters

**2.2** Hidden Algorithmic Parameters

**3. Job Processing**

**4. CensuScope Results**

**INTRODUCTION**

The HIVE **CensuScope** tool determines the taxonomic composition in metagenomic sample by analyzing the sequence data through rapid iterative mapping to all known sequences using BLAST or HIVE-hexagon. This tutorial assumes data has already been loaded into HIVE using HIVE’s **dmDownloader** utility. For more information, please see the document titled “HIVE Downloader Tutorial” available in main pages of HIVE website. CensuScope can be used for quick sample origin discovery, to study metagenomic samples or mixed viral populations, or to evaluate the possible contamination of samples. CensuScope result is downloadable in SVG format (for Taxonomy tree) and CSV format for the mapping results.

**1. SELECTING INPUTS**

Once logged into HIVE user home directory, select your desired input read file by clicking on its row in the table. Appropriately selected objects will be displayed with the row containing it highlighted in purple. After selecting an object, new tools will appear in the toolbar immediately above the directory (See Figure 1)

**Figure 1. Selecting CensuScope from Toolbar**

**
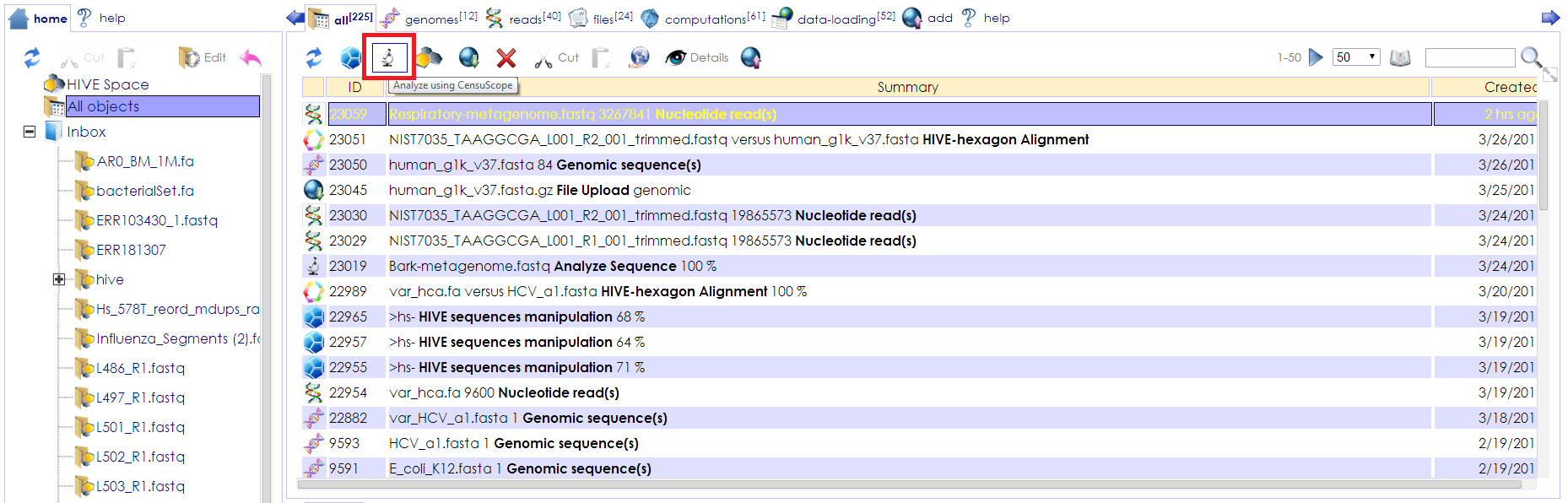
**

Clicking on the
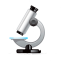
 **CensuScope** icon will direct you to the CensuScope algorithmic portal.

**2. INPUT PARAMETERS**

The CensuScope tool page (See Figure 2) is organized such that the top **CensuScope** box automatically displays the most frequently modified and required parameters, with additional parameters available by clicking the expansion
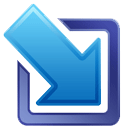
 icon to the left of the **Parameters** section header. To hide the section when open, click the
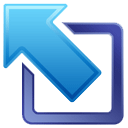
 icon to the left of the section header.

**Figure 2. CensuScope Tool Portal**


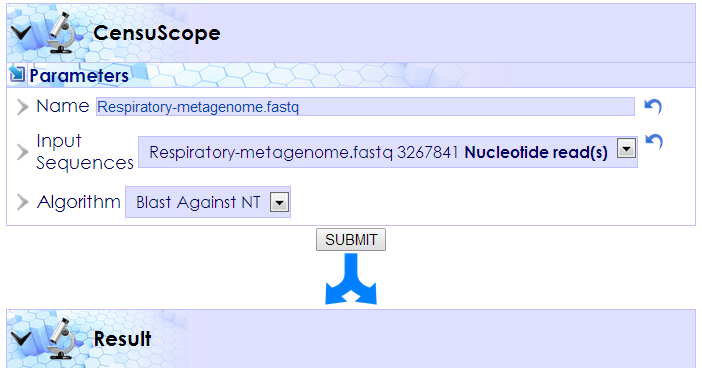


- 1. **Most frequently modified parameters**

**Name:** Specify a name for the process. If the user does not enter a name, HIVE will automatically name it using the input file name.

**Input Sequences:** This should display the sequence (or sequences) selected from the user home directory. To select additional input reads, click the arrow for the dropdown menu. A pop-up replica of your user home directory will appear. Select multiple files by clicking and highlighting each one. To close click the
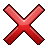
 close icon in the top right corner.

**Algorithm:** Select which alignment algorithm to use to query the NT database.

**HIVE-hexagon aligner** was developed by HIVE team and as such natively uses HIVE’s parallel execution architecture resulting in improved speed, sensitivity and accuracy of alignments compared with current methods.

**BLAST** is the Basic Local Alignment Search Tool maintained by NCBI at NLM and is a widely used tool for calculating sequence similarity. BLAST has been adapted to run optimally in HIVE’s highly parallel environment, taking full advantage of HIVE’s distributed data storage through explicit parallelization.

**Bowtie2** is a popular mapping algorithm that is faster than BLAST.

**Reference databases:** Select which database to search against.

**BLAST-NT** contains all publicly available nucleotide sequences from NCBI.

**MetaPhlAn** is a signature database of bacterial origin available from MetaPhlAn team.

**Representative Genomes** database consists of sequences from selected organisms.

- 1. **Hidden Algorithmic Parameters**

More customizable parameters can be viewed by clicking the expansion button
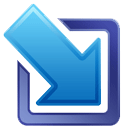
 found on the top left corner of the **Parameters** box.

**Iteration:** Default set to 1**0**.

This process is repeated multiple times to ascertain the taxonomic composition that is found in majority of the iterations

**Sample Size:** Default set to 1**000**.

Number of sequence reads to analyze

**Taxonomy Depth:** Default set to **3**.

Level of nodes within a given taxonomy tree/system

**3. JOB PROCESSING**

Click the **SUBMIT** button to start the job. The page will be refreshed and a new box will appear in the middle of the page tracking the progress of the task (Figure 3.)

By clicking on the expand node icon
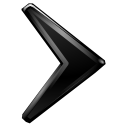
 found in the top left side of this box, you can view the progress of every subcomponent of this task. Once 100% complete, the process status will change from **Waiting** to **Running** to **Done.** The entire process is complete when all statuses read **Done** and the progress bar reads **100% completion**. Results will populate the bottom **Result** section when complete

**Figure 3. Tracking CensuScope Progress**

**
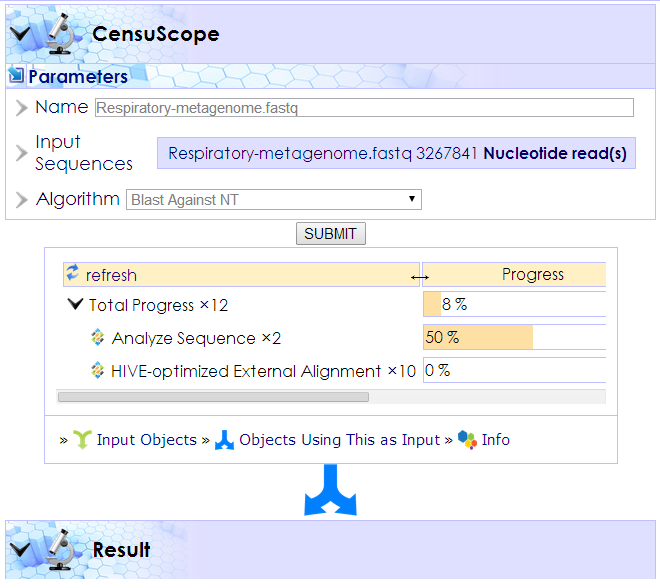
**

**4. CENSUSCOPE RESULTS**

The **CensuScope** results box has two components: the left summary viewer and the right detailed visualization viewer (Figure 4).

The default view of **CensuScope** results is the **phylogram** view containing partially-collapsed taxonomic hierarchies of all species found in the sample by CensuScope computations. Also available is a
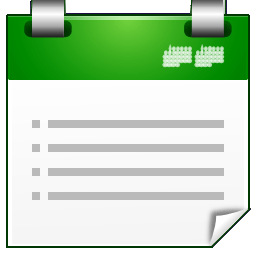
 **list** view which summarizes taxonomy hits in a table by taxonomy ID, organism name, number of hits to the relevant ID and number of taxonomic children to the taxonomy ID present in the sample (Figure5).

To view detailed taxonomy information about a specific organism, either click the node (can be species or higher level hierarchical node) from the phylogram representation or select the containing row from the list view. Once selected, taxonomy information about the selected species will now populate the right box of the results section. Information displayed includes, when available: Taxonomy nodes, Bio Project ID, Common names, Taxonomy names, Parent rank and Taxonomy ID

**Figure 4. CensuScope Results Taxonomy tree View**

**
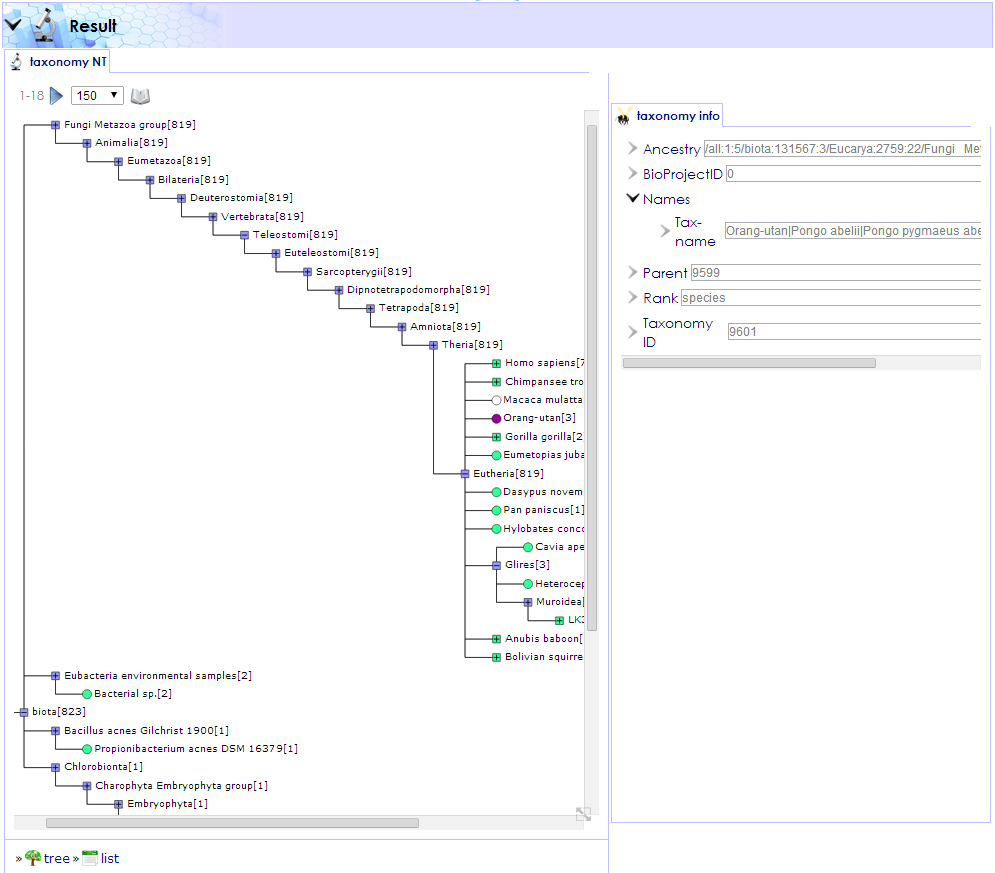
**

**Figure 5. CensuScope Results List View**

**
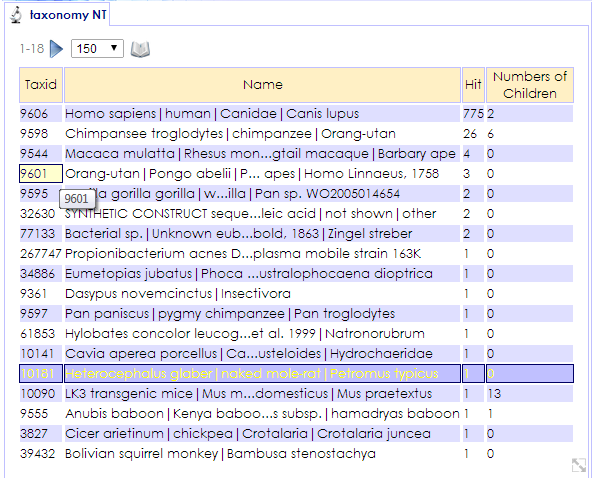
**
